# Pre- and post-LEEP: analysis of the female urogenital tract microenvironment and its association with sexual dysfunction

**DOI:** 10.1093/sexmed/qfad039

**Published:** 2023-08-14

**Authors:** Olivia Giovannetti, Diane Tomalty, Leah Velikonja, George Gray, Nadejda Boev, Shelby Gilmore, Jummy Oladipo, Calvin Sjaarda, Prameet M Sheth, Michael A Adams

**Affiliations:** Department of Biomedical and Molecular Science, Queen’s University, Kingston K7L3N6, Canada; Department of Biomedical and Molecular Science, Queen’s University, Kingston K7L3N6, Canada; Department of Biomedical and Molecular Science, Queen’s University, Kingston K7L3N6, Canada; Department of Obstetrics and Gynaecology, Kingston General Hospital, Kingston K7L3N6, Canada; Department of Pathology and Molecular Medicine, Queen’s University, Kingston K7L3N6, Canada; Department of Biomedical and Molecular Science, Queen’s University, Kingston K7L3N6, Canada; Department of Biomedical and Molecular Science, Queen’s University, Kingston K7L3N6, Canada; Department of Pathology and Molecular Medicine, Queen’s University, Kingston K7L3N6, Canada; Department of Biomedical and Molecular Science, Queen’s University, Kingston K7L3N6, Canada; Department of Pathology and Molecular Medicine, Queen’s University, Kingston K7L3N6, Canada; Department of Biomedical and Molecular Science, Queen’s University, Kingston K7L3N6, Canada

**Keywords:** microbiota, cervix uteri, vagina, colposcopy, uterine cervical dysplasia, sexual dysfunctions

## Abstract

**Background:**

The loop electrosurgical excision procedure (LEEP) to treat cervical dysplasia (CD) is known to alter the cervical microbiota, the community of bacteria that play a central role in female genital health. Perturbations to the microbiota of the female urogenital tract (FUT), including the urethra, vagina, and cervix, have been linked with symptoms of sexual dysfunction (SD), though correlations among LEEP, the microenvironment, and SD have not yet been described.

**Aims:**

To characterize the FUT microbiota before and after LEEP and investigate possible associations with SD.

**Methods:**

Females undergoing LEEP for CD were recruited to participate in the study. Urinary samples and vaginal and cervical swabs were collected immediately before and 3 months after treatment. Bacterial communities were characterized by 16S rRNA next-generation sequencing. Self-report surveys assessing demographics, medical history, and sexual function were completed at the same intervals.

**Outcomes:**

Microbiota taxonomy and Female Sexual Function Index (FSFI) scores.

**Results:**

Alpha diversity revealed a significant decrease in species richness in the FUT microbiota post-LEEP. Beta diversity demonstrated significant differences among the cervical, urinary, and vaginal microenvironments pre- and post-LEEP. *Lactobacillus* spp were the dominant microbial genus in the cervical microenvironment pre- and post-LEEP. Although the vaginal and urinary microenvironments were characterized by *Prevotella* pre-LEEP, they were colonized by *Lactobacillus* post-LEEP. Following LEEP, some participants experienced a significant increase in proinflammatory bacteria, including the genera *Gardnerella*, *Megasphaera*, *Sneathia*, *Parvimonas*, and *Peptostreptococcus.* Others experienced significant decreases in inflammatory and protective bacteria post-LEEP, including *Butyricicoccus*, *Terriporobacter*, *Intestinimonas*, and *Negativibacillus.* Overall there were no significant changes in pre- and post-LEEP FSFI scores. However, post-LEEP FSFI scores were seemingly associated with changes in inflammatory bacteria in some participants.

**Clinical Implications:**

There is an overall reduction in FUT microbiota dysbiosis post-LEEP. However, we show variability as some participants experienced persistent dysbiosis of FUT microbiota and elevated FSFI scores, suggesting that therapies to treat dysbiosis of FUT microbiota may reduce FSFI scores, thereby improving SD symptoms.

**Strengths and Limitations:**

We demonstrate novel associations among urogenital sites, microbiota changes, LEEP, and SD. The small sample size and inability of species classification are limitations.

**Conclusion:**

Diverse inflammatory microbiota characterizes CD in the FUT, and LEEP mostly returns microenvironments to a healthy state. However, some participants have persistent inflammatory bacteria post-LEEP, suggesting a non-uniform healing response. This study provides an impetus for future longitudinal studies to monitor and restore FUT microenvironments post-LEEP, aimed at mitigating postoperative SD symptoms.

## Introduction

The female urogenital tract (FUT) is a microenvironment that is constantly changing in response to internal and external stimuli. The FUT is composed of the urethra, vagina, and cervix, and the anatomic sites along the FUT have largely been studied independently^1-3^ or in pairs.^4^^,^^5^ Therefore, the entire FUT has yet to be investigated. Given their anatomic proximity and function, it is important to understand the characteristic bacterial profiles of these three regions in parallel to assess any symbiotic relationships. Clinically, there is evidence linking altered bacterial profiles to certain disease states, such as bacterial vaginosis,^6^ endometriosis,^5^ urinary tract infections,^7^ and sexually transmitted infections.^8^ However, there has been limited work to date characterizing the microbiota related to diseases of the cervix.^9^^,^^10^

With regard to bacteria populations, the cervix and vagina are distinct.^1^ Given their proximity, however, they are often investigated as a single “cervicovaginal” environment and characterized as having a predominance of *Lactobacillus*.^11^ Likewise, the urethra and vagina have similar cellular and bacterial populations,^4^^,^^12^ and both are also dominated by lactobacilli.^13^*Lactobacillus* has been associated with an anti-inflammatory cervicovaginal immune profile; it has also been shown to protect women from developing anaerobic dysbiosis and to reduce the risk of acquiring sexually transmitted infections.^14^ Currently, the vaginal microenvironment has been studied far more than the other two regions of interest along the FUT, especially regarding the symptomatic effects of dysbiosis. Vaginal dysbiosis can be a result of a high-diversity, lactobacilli-deficient microenvironment.^3^^,^^14^ Adverse outcomes from vaginal dysbiosis includes odor, vaginal discharge, and irritative symptoms such as itching and pain.^15^ Together, these symptoms can reduce an individual’s quality of life^16^ and are associated with decreased sexual function.^17^

Cervical dysplasia (CD) is a common gynecologic condition that affects hundreds of thousands of women annually.^18^ CD is most commonly caused by human papilloma virus (HPV) infections and manifests as abnormal cells on the cervix.^9^ While efforts have been made to characterize cervicovaginal bacterial communities associated with HPV infection,^19-21^ little is known about the microenvironment of the general patient population with CD. Treatments for CD, such as the loop electrosurgical excision procedure (LEEP), have been found to affect the cervical^10^^,^^22^ and vaginal^22^ microenvironments with potential to alter the urinary tract.^23^ LEEP excises abnormal cells of the cervix via a heated wire loop and is considered the gold standard treatment for CD^24^; however, these reported effects demonstrate the need for a complete FUT microenvironment study in relation to this procedure.

Furthermore, CD treatments have been associated with sexual impairments^25-28^ and psychosexual sequelae^29^^,^^30^ in some patients, and correlations between these symptoms and the FUT microenvironment should be investigated. Specifically, negative outcomes have been reported, such as decreased sexual function,^25-28^ lubrication,^25^ arousal,^25^^,^^26^ orgasm frequency,^25^ orgasm/sexual satisfaction,^27^ and increased dyspareunia.^25^ Limited evidence has implicated cervical dysbiosis in the pathogenic progression of HPV-induced cervical changes—the precursor to CD.^9^ Although one study showed changes in the dominant bacterial population characterizing the cervical microenvironment after LEEP treatment,^10^ the patients in this study were not assessed for symptoms of sexual dysfunction (SD).

The present study aimed to answer the following questions: (1) Which microbiota characterize the urinary, vaginal, and cervical environments in patients with CD before and after treatment with LEEP? (2) Are there possible associations of microbiota with self-reported symptoms of SD?

## Methods

### Study design

This was a prospective study evaluating the FUT microbiota in patients with CD undergoing LEEP treatment at the Colposcopy Clinic at Kingston Health Sciences Centre, Kingston, Canada, from November 2020 to November 2021. The study was approved by the Queen’s University Health Sciences and Affiliated Teaching Hospitals Research Ethics Board, and all participants provided written informed consent.

### Sample collection

Participants had previously undergone pelvic examinations with clinical inspection for CD cervical intraepithelial neoplasia (CIN) 2/3; and were referred to the Colposcopy Clinic for treatment by LEEP. At the treatment visit, participants provided a urine sample immediately before surgery. Clinicians then inserted a speculum in preparation for LEEP and collected a midvaginal swab and a cervical swab. Samples were frozen at −80 °C immediately after collection until analysis.

### DNA extraction

To prepare for DNA extraction, all samples were thawed and vortexed for 1 minute to homogenize the solutions. DNA was isolated with the DNeasy PowerSoil Kit (Qiagen) according to the manufacturer’s instructions.

### Next-generation sequencing

#### 16S rRNA sequencing

The V3 and V4 regions of the 16S rRNA gene were amplified in a 50-μL polymerase chain reaction with Invitrogen Platinum Taq DNA Polymerase:

Forward primer: 5′-TCGTCGGCAGCGTCAGATGTGTATAAGAGACAGCCTACGGGNGGCWGCAG-3′

Reverse primer: 5′- GTCTCGTGGGCTCGGAGATGTGTATAAGAGACAGGACTACHVGGGTATCTAATCC-3′

Libraries were constructed and barcoded by the Nextera DNA Library Preparation Kit (FC-121-1011; Illumina). DNA amplicon purity and concentration were quantified on a 2100 BioAnalyzer (Agilent Technologies) and Qubit 3.0 Fluorometer (Thermo Fisher Scientific). Finally, the barcoded samples were normalized to equal concentration, pooled, and then sequenced with the MiSeq Sequencer (Illumina).

#### Sequence data processing

Raw sequencing read quality was assessed with the tool FastQC (version 0.11.9). Low-quality nucleotides and adapter sequences were removed with Cutadapt (version 3.4) within the TrimGalore (version 0.6.6) wrapper. Reads were processed with DADA2 for quality filtering (a 15-bp left trim and forward and reverse reads were trimmed at 280 and 250 bases, respectively), denoising, and chimera removal.^31^ Reads were annotated and compared against a Naïve Bayesian classifier with the 2020 version of the Ribosomal Database Project. Finally, we rarefied to 10,000 reads.

### Survey distribution

Participants completed an online questionnaire immediately before their LEEP and at their 3-month follow-up appointment. The survey collected detailed information on demographics, gynecologic and relevant medical history, sexual health, barriers to discussing sexual concerns, and sexual functioning. Additionally, participants completed the Female Sexual Function Index (FSFI),^32^ a 19-item questionnaire assessing sexual function over the past 4 weeks.

### Statistical analysis

We calculated alpha and beta diversity—quantified by observed species and Shannon index and by Bray-Curtis, respectively—using the phyloseq (version 1.34.0)^33^ and vegan (version 2.5-7)^34^ packages in R (version 4.0.2).^35^ Relative abundance of bacteria across different regions by time point was analyzed via Welch *t* tests. Relative abundance of bacteria per participant was calculated with agglomerated compositions of time points and regions via analysis of variance (ANOVA). We visualized and analyzed beta diversity with principal components analysis (PCA), whereby ellipses were drawn at a 95% confidence interval (CI) and clusters were compared with permutational multivariate analysis of variance (PERMANOVA). When comparing bacterial abundances for individuals pre- and post-LEEP, we used a paired *t* test.

Survey data were analyzed with SPSS version 26 (IBM). Only participants with complete questionnaire data were included in the analyses (N = 23). The significance level for all tests was set at an α of 0.05. Independent *t* tests were conducted to assess differences between FSFI scores pre- and post-LEEP.

## Results

### Participant characteristics

Twenty-five participants were recruited and consented to participate in this study. Two individuals without cervical, urinary, and vaginal samples were excluded from the study. The basic demographic and health characteristics of the remaining 23 participants are presented in Table 1. One of these participants did not provide a vaginal sample. Most participants were between the ages of 21 and 30 years, identified as heterosexual, were premenopausal, and almost always or always had sexual activity without barrier use. Sixty percent of participants had previously undergone a gynecologic procedure, most commonly a cervical cone biopsy. No participants disclosed that they were being treated for pelvic or genital infection during the study period.

**Table 1:** Demographic characteristics and health history (N = 23).

**Variable**	**No. (%)**
Age, y	
21-30	15 (65.2)
31-40	3 (13.0)
41-50	2 (8.7)
≥51	3 (13.0)
Sexual orientation	
Heterosexual	20 (87.0)
Bisexual	3 (13.0)
Menopausal status	
Premenopausal	18 (78.3)
“I don’t know”	5 (21.7)
Gynecologic procedure	
LEEP	2 (8.7)
Cervical cone biopsy	6 (26.1)
Laser ablation of cervix	2 (8.7)
Caesarian section	2 (8.7)
Other	2 (8.7)
NA	9 (39.1)
Frequency of sexual activity without barrier use	
Almost never or never	3 13.0)
Sometimes	4 (17.4)
Most times	1 (4.4)
Almost always or always	15 (65.2)
Hormonal medication	
Birth control	11 (47.8)
None	12 (52.2)

Abbreviations: LEEP, loop electrosurgical excision procedure; NA, not applicable.

### Sequence summary

A total of ~3 million high-quality reads were obtained with an average of 30 998 per cervical sample, 15 943 per urinary sample, and 22 700 per vaginal sample. The mean number of genera observed was 36.2 in the cervical microenvironment, 58.5 in the urinary, and 67.5 in the vaginal. Low abundance genera (<1% of total reads) were filtered for visualization.

### Bacterial richness and diversity differ between regions of the FUT and differ pre- and post-LEEP

**Figure 1:**
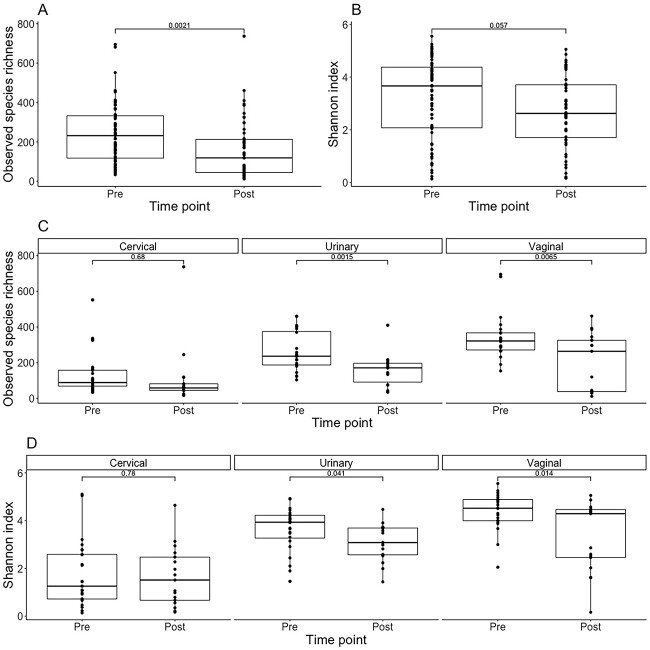
Alpha diversity of FUT pre- and post-LEEP. (A) Observed species richness among all regions of the FUT pre- and post-LEEP. (B) Diversity assessed by Shannon index among all regions of the FUT pre- and post-LEEP. (C) Observed species richness of the cervical, urinary, and vaginal microenvironments pre- and post-LEEP. (D) Shannon index of the cervical, urinary, and vaginal microenvironments pre- and post-LEEP. FUT, female urogenital tract; LEEP, loop electrosurgical excision procedure.

We found that alpha diversity, as measured with observed species richness (*P* = .0021) and Shannon index (*P* = .057), demonstrated a decrease in diversity post-LEEP (Figure 1A and 1B). Anatomic site–specific analysis revealed that the urinary and vaginal microenvironments experienced significant decreases in species richness post-LEEP (*P* = .0015 and *P* = .0065, respectively; Figure 1C). In contrast, there were no changes detected in the species richness of the cervical microenvironment (*P* = .68; Figure 1C). This was confirmed by observed species richness and the Shannon index (Figure 1C and 1D).

**Figure 2:**
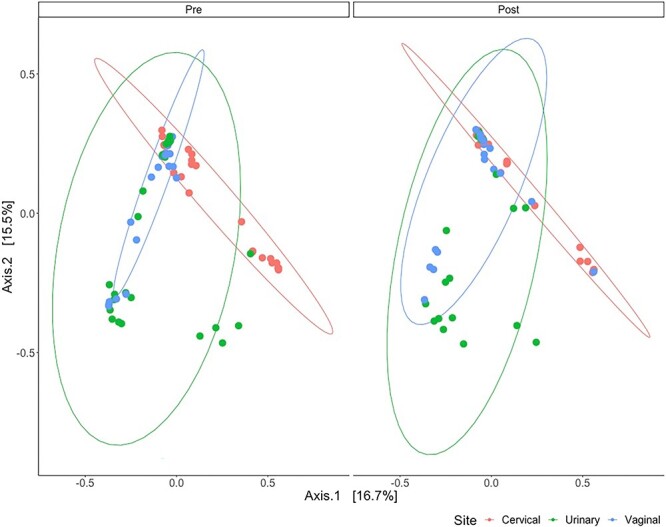
Beta diversity of the cervical, urinary, and vaginal microenvironment pre- and post-LEEP via principal components analysis, calculated by Bray-Curtis dissimilarity. LEEP, loop electrosurgical excision procedure.

Furthermore, to investigate the beta diversity among regions of the FUT and pre- and post-LEEP, we applied principal components analysis (Figure 2). Results from pairwise PERMANOVA demonstrated that beta diversity significantly differed among all regions of the FUT pre- and post-LEEP. This included the cervical microenvironment, as it was observed to be significantly different from the vaginal microenvironment pre-LEEP (*P* = .001) and post-LEEP (*P* = .008) and the urinary microenvironment pre-LEEP (*P* = .001) and post-LEEP (*P* = .002). Additionally, the urinary microenvironment was significantly different from the vaginal microenvironment pre-LEEP (*P* = .006) and post-LEEP (*P* = .046). The vaginal microenvironment itself significantly changed post-LEEP (*P* = .009); however, we found no significant changes when comparing within the clusters of the cervical (*P* = .983) and urinary (*P* = .065) microenvironments pre- and post-LEEP (Figure 2).

### Bacterial composition differs among regions of the FUT and pre- and post-LEEP

**Figure 3:**
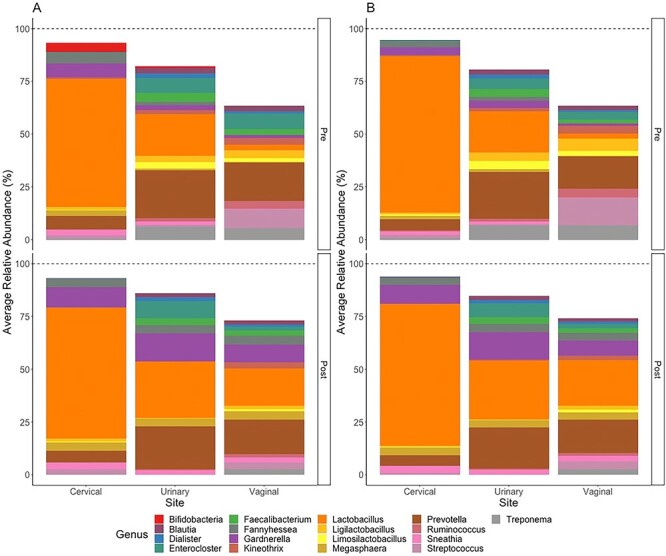
Average cervical, urinary, and vaginal microenvironments of participants pre- and post-LEEP at the genus level. (A) All participants. Pre-LEEP, n = 22; post-LEEP, n = 17. (B) Pre-LEEP average relative abundance does not include participants lost to follow-up (n = 17); post-LEEP, n = 17. Unassigned bacteria not included. LEEP, loop electrosurgical excision procedure.

Upon rarefaction, 120 genera were observed in the cervical microenvironment, 111 in the urinary, and 93 in the vaginal. A total of 324 unique bacteria genera were observed among all regions of the FUT, with the 17 most abundant bacteria genera listed in Figure 3. Figure 3 also illustrates the average cervical, urinary, and vaginal microenvironments pre- and post-LEEP for all participants (n = 22 and 17, respectively) and those with paired samples (n = 17). We found that *Lactobacillus* sp was the most common bacteria genera in the cervix given its highest relative abundance pre- and post-LEEP. In the urinary microenvironment, *Lactobacillus* and *Prevotella* were observed to be highly abundant pre- and post-LEEP, in which both bacteria had an average relative abundance >20%. However, in the urinary microenvironment, we observed a decrease in the relative abundance of *Prevotella* post-LEEP (Figure 3A). The average vaginal microenvironment pre-LEEP demonstrated a low relative abundance of *Lactobacillus*, with a higher abundance of *Prevotella* and *Streptococcus*, although >25% of the average bacterial composition was composed of bacteria with low read counts or unassigned bacteria. Post-LEEP, the average vaginal microenvironment demonstrated decreased *Streptococcus* and enriched *Lactobacillus* (Figure 3A). The same results were observed for participants with complete samples (Figure 3B).

### Relative abundance of bacterial genera differs among regions of the FUT and pre- and post-LEEP

**Figure 4:**
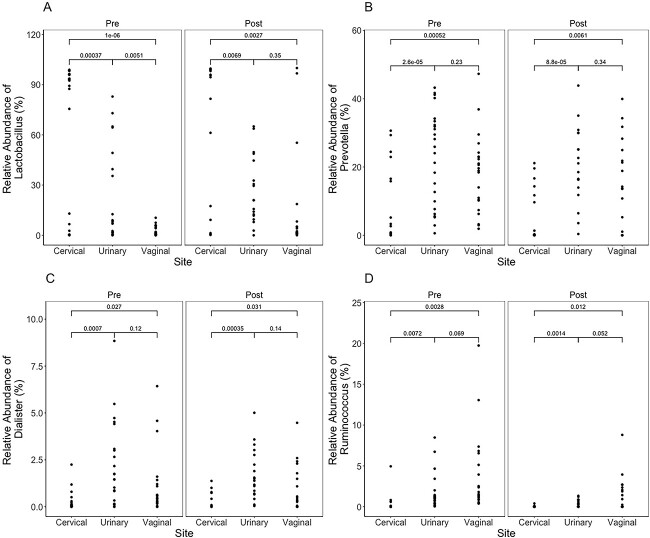
Relative abundance (percentage) between regions of the female urogenital tract microenvironment pre- and post-LEEP for *Lactobacillus* (A), *Prevotella* (B), *Dialister* (C), and *Ruminococcus* (D). LEEP, loop electrosurgical excision procedure.

Spatially, the FUT microenvironment displayed differences in numerous bacteria genera among regions, including *Lactobacillus*, *Prevotella*, *Dialister*, and *Ruminococcus* (Figure 4). The cervical microenvironment pre- and post-LEEP demonstrated differing abundances of *Lactobacillus* as compared with the urinary (*P* = .00037, *P* = .0069, respectively) and vaginal (*P* = 1 × 10^−6^, *P* = .0027) microenvironments. The urinary and vaginal microenvironments were also observed to be significantly different pre-LEEP (*P* = .0051), although post-LEEP results showed no significant differences between the two locations the two locations (*P* = .35). The cervical microenvironment additionally revealed a significantly lower relative abundance of *Prevotella*, *Dialister*, and *Ruminococcus* when compared with the urinary microenvironment pre-LEEP (*P* = 2.6 × 10^−5^, *P* = .0007, *P* = .0072) and post-LEEP (*P* = 8.8 × 10^−5^, *P* = .031, *P* = .0014), as well as the vaginal microenvironment pre-LEEP (*P* = .0005, *P* = .0004, *P* = .0028) and post-LEEP (*P* = .0061, *P* = .031, *P* = .12). However, no significant differences were observed between urinary and vaginal microenvironments.

**Figure 5:**
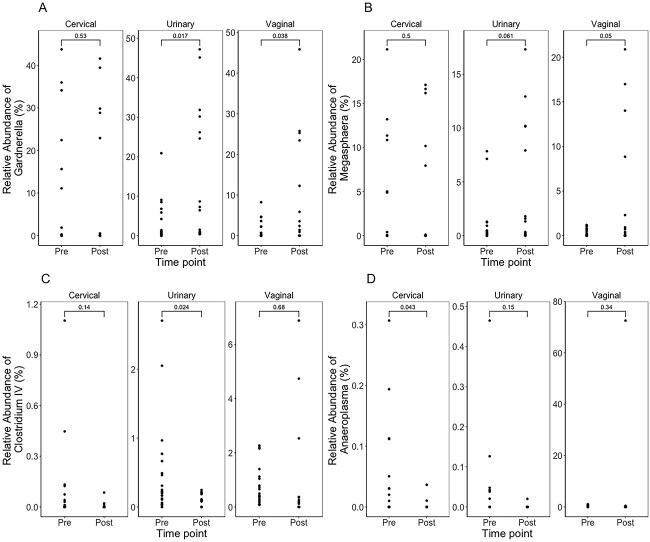
Relative abundance (percentage) between pre- and post-LEEP samples from the cervical, urinary, and vaginal microenvironments for *Gardnerella* (A), *Megasphaera* (B), *Clostridium* (C), and *Anaeroplasma* (D). LEEP, loop electrosurgical excision procedure.

Temporally, we noted variable increases and decreases in relative abundance by FUT region, pre- and post-LEEP (Figure 5). *Gardnerella* was observed to significantly increase in the urinary and vaginal microenvironments post-LEEP (*P* = .017 and *P* = .038, respectively; Figure 5A). Although not statistically significant, *Megasphaera* increased post-LEEP in the urinary and vaginal microenvironments (Figure 5B). In the urinary microenvironment, *Clostridium* significantly decreased post-LEEP (*P* = .024; Figure 5C). Last, the cervical microenvironment displayed a significant decrease in the relative abundance of *Anaeroplasma* (*P* = .043; Figure 5D).

### Participants experience altered relative abundance of bacteria genera pre- and post-LEEP

When the FUT was observed pre- and post-LEEP, most participants had a cervical microenvironment dominated by *Lactobacillus*, with more diversity in the urinary and vaginal microenvironments. We found that individuals showed a high degree of variance in their bacterial composition, spatially and temporally. For example, only some participants experienced an increase in the relative abundance of *Lactobacillus* post-LEEP. Among all regions of the FUT, the relative abundance of *Lactobacillus* and *Prevotella* remained relatively constant pre- and post-LEEP. Of the 14 participants with complete samples, 6 had an increase in the relative abundance of *Gardnerella* post-LEEP.

**Figure 6:**
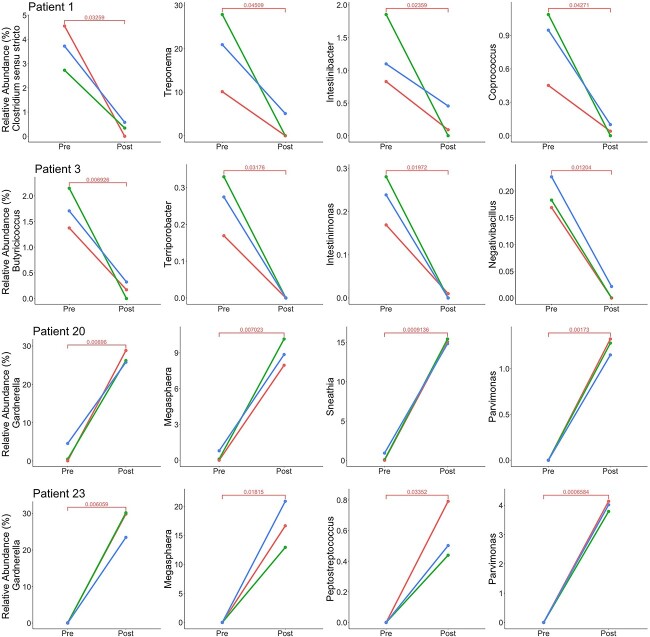
Relative abundance (percentage) pre- and post-LEEP of various bacteria for participants 1, 3, 20, and 23. Bacteria experienced a significant change pre- and post-LEEP among all regions of the FUT microenvironment. FUT, female urogenital tract; LEEP, loop electrosurgical excision procedure.

A subanalysis of individual participant data revealed some participants who experienced significant changes in bacteria associated with dysbiosis (Figure 6). Post-LEEP, participant 1 had significant decreases in *Clostridium sensu stricto* (*P* = .0325), *Treponema* (*P* = .0459), *Intestinibacter* (*P* = .0235), and *Coprococcus* (*P* = .0427). A significant decrease was also observed post-LEEP in the FUT microenvironment of participant 3 for *Butyricicoccus* (*P* = .0069), *Terriporobacter* (*P* = .0317), *Intestinimonas* (*P* = .0197), and *Negativibacillus* (*P* = .0120). Conversely, participants 20 and 23 experienced an increase in bacteria associated with FUT dysbiosis. Participant 20 had a significant increase in *Gardnerella* (*P* = .0069), *Megasphaera* (*P* = .0072), *Sneathia* (*P* = .0009), and *Parvimonas* (*P* = .0017) post-LEEP. Participant 23 also had a significant increase in various bacteria post-LEEP, including *Gardnerella* (*P* = .006), *Megasphaera* (*P* = .0181), *Peptostreptococcus* (*P* = .0335), and *Parvimonas* (*P* = .0006).

### Individual participant bacterial compositions were aligned with survey responses

To investigate whether bacterial composition is associated with SD, survey responses retrieved from FSFI questionnaires were analyzed. The FSFI has a maximum score of 36 and a threshold of 26.55, whereby scores below threshold may indicate SD. Participant 1 displayed a slight decrease in FSFI score, from 34.5 pre-LEEP to 32.7 post-LEEP, though their scores remained high. In contrast, participant 3 experienced a purported increase in sexual functioning, with a score of 21.9 pre-LEEP to 34.4 post-LEEP, with reported improvements in desire, arousal, lubrication, satisfaction, and pain domains. Based on their scores, participants 20 and 23 indicated SD both pre- and post-LEEP. Participant 20 had the same FSFI score of 24.5 pre- and post-LEEP, with a minor increase in arousal and orgasm but a decrease in lubrication and satisfaction. Participant 23 experienced a decrease in overall FSFI score from 25 pre-LEEP to 23.6 post-LEEP, due to decreases in the domains of arousal and lubrication; however, they did experience in increase in orgasm. Possible associations between the reported bacterial composition of these 4 participants and their FSFI scores are presented in Table 2.

**Table 2:** Possible associations between participants’ bacterial composition and FSFI scores.

	**FSFI overall scores**		**FSFI domain changes**		**Significant bacteria changes**		
**No.**	**Pre-LEEP**	**Post-LEEP**	**Decrease**	**Increase**	**Decrease**	**Increase**	**Functional roles of bacteria**
1	34.5	32.7	Desire, orgasm, pain	—	*Clostridium sensu stricto*, *Treponema*, *Intestinibacter*, *Coprococcus*	—	Intestinal inflammation, genital infections, gut dysbiosis, butyrate producer (protective)
3	21.9	34.4	—	Desire, arousal, lubrication, satisfaction, pain	*Butyricicoccus*, *Terriporobacter*, *Intestinimonas*, *Negativibacillus*	—	Mucosa-associated butyrate producer, microbial infection, butyrate producer, inflammation
20	24.5	24.5	Lubrication, satisfaction	Arousal, orgasm	—	*Gardnerella*, *Megasphaera*, *Sneathia*, *Parvimonas*	Epithelial damage, vaginal infection, pathogen obstetric complications, genital mucosa injury infection
23	25	23.6	Arousal, lubrication	Orgasm	—	*Gardnerella*, *Megasphaera*, *Peptostreptococcus*, *Parvimonas*	Epithelial damage, vaginal infection, HPV infection, genital mucosa injury infection

Abbreviations: FSFI, Female Sexual Function Index; LEEP, loop electrosurgical excision procedure.

### Female sexual functioning pre- and post-LEEP

Upon analysis of the FSFI for all participants, no significant changes were observed to female sexual functioning overall pre- and post-LEEP (*P* > .05). The mean FSFI score was 29.13 ± 4.19 pre-LEEP and 28.75 ± 4.07 post-LEEP (*P* = .884). Additionally, no significant changes were observed in any domains, including desire, arousal, lubrication, orgasm, satisfaction, and pain. For example, the mean lubrication score pre-LEEP was 5.32 ± 0.89 and 5.24 ± 0.72 post-LEEP (*P* = .554).

## Discussion

This study is the first to demonstrate a decrease in overall microbiota dysbiosis post-LEEP for the cervical, urinary, and vaginal microenvironments in most participants, as determined by a decrease in species richness and change in the relative abundance of certain bacteria (eg, *Lactobacillus*, *Streptococcus*, *Prevotella*, *Dialister*, and *Ruminococcus*). Results also determined interrelatedness among the FUT microbiota, as variability was observed among the cervical, urinary, and vaginal microenvironments, though they largely comprised similar bacteria. Additionally, by examining the microbial profile by individual, we potentially revealed an uneven healing response post-LEEP. That is, we observed that a subset of patients had increases in proinflammatory bacteria and persistent SD post-LEEP, while others had decreases in proinflammatory bacteria and improved FSFI scores post-LEEP. This study contributes to the growing body of evidence characterizing the cervix in patients with CD and provides a basis for future investigation of therapies to address persistent dysbiosis and associated bothersome symptoms related to sexual function.

There have been several recent studies of the cervicovaginal^36^ and individual vaginal^37-39^ and cervical^40^^,^^41^ microenvironments in relation to HPV infection and cervical cancer development. We contributed to this work by focusing on FUT microbiota in patients with CD diagnoses that had not progressed to cervical cancer. Historically, the cervicovaginal microenvironment has shown to be dominated by *Lactobacillus*.^42^ Our findings align with reports, as these data reveal *Lactobacillus* abundance in the pre-LEEP (CD) cervix microenvironment. In the vagina, *Prevotella* and *Streptococcus* were more abundant than *Lactobacillus* pre-LEEP, which may show an inverse relationship in this disease state. The urinary microenvironment showed *Lactobacillus* and *Prevotella* as most abundant pre-LEEP, revealing some similarity to the cervical and vaginal compositions. These findings support other studies that have demonstrated an inverse relationship between cervical disease and *Lactobacillus-*dominant environments in FUT regions.^23^ Our results may also indicate an interrelatedness among these 3 regions within the FUT, which supports current evidence.^11^^,^^13^

Given that our study investigated FUT regions separately, we found a potentially unique connection between the urethra and the vagina and cervix, providing new evidence that the urogenital tract could be considered when evaluating CD pathogenesis, instead of solely the genital tract.^37-39^ Understanding the interplay and cross-communication among bacteria in these 3 regions could be used to investigate less invasive testing^43^ for clinical indicators,^36^ such as urine samples^44^ or vaginal swabs.^45^ There are, however, several variables that can affect urinary and reproductive tract microenvironments and limit interpretation. These include menstrual cycle, oral contraceptives, stress, and sexual activities,^46^ which should be explored in future analyses.

A major focus of this study was the analysis of FUT microenvironment regions in a disease state, pre-LEEP, as compared with a nondisease state, post-LEEP. While the cervical and vaginal microenvironments have been investigated in patients with CD,^36^^,^^37^^,^^41^ few studies have studied the effects of treatments such as LEEP in this capacity.^10^^,^^22^ It has been reported that LEEP appears to alter the cervix to a less diverse microbiota^10^^,^^22^ and promote the restoration of a *Lactobacillus*-dominant community,^10^ which aligns with the findings in this study. However, our data showed that the cervix microenvironment was *Lactobacillus* dominant pre- and post-LEEP upon analysis at the genus level. As the limited evidence to date is reported from studies with small samples sizes,^10^^,^^22^ confirmatory investigations should be done to elucidate changes pre- and posttreatment in the cervix.

Yet, this study did detect changes in relative abundance post-LEEP in the vagina and urethra, identifying decreases in *Streptococcus* and *Prevotella*, respectively, and a return to *Lactobacillus* dominance. Despite these changes, the overall microenvironment of the FUT remained largely the same pre- and post-LEEP. These findings show that patients with CD have FUT dysbiosis,^47^ which seems to be restored by LEEP treatment. Nevertheless, given that the FUT microenvironment is dynamic and is thus highly susceptible to change,^48^ we further investigated changes experienced by individual participants.

While the overall FUT appeared relatively stable pre- and post-LEEP, analysis of individual participant bacterial composition showed that a subset of participants (6/14) had increases in *Gardnerella* post-LEEP. These findings deviate from the narrative that describes most participants, where healthy microbiota are restored.^10^^,^^22^ We were further interested in the variability among individuals and identified 4 participants who had significant changes in bacteria genera pre- and post-LEEP. Participants 1 and 3 had significant decreases in proinflammatory and protective bacteria. These changes may be characteristic of a normalized healing response, with decreases in these potentially harmful bacteria.^49-53^ Comparatively, a decrease in protective bacteria,^54-56^ especially in the case of butyrate producers that can attenuate inflammation, could mean that these were upregulated pre-LEEP in a diseased inflammatory state.^57^ Their significant decrease could indicate a return to normal in these participants.

In contrast, participants 20 and 23 had significant increases in pathogenic bacteria, including 75% of the same bacteria that significantly increased post-LEEP. All bacteria have been previously associated with cervicovaginal infection,^58-61^ cellular damage,^60^^,^^62^ or harmful reproductive outcomes.^58^^,^^59^ Specifically, *Sneathia* and *Gardnerella* have been associated with CD lesion progression,^36^^,^^63^^,^^64^ and *Gardnerella* has been associated with slow infection resolution.^19^ Our findings suggest that these other bacteria may be associated with the CD microenvironment, specifically in the case of this excisional treatment. While conclusions cannot be drawn from such a small subset of participants, it is possible that these bacteria provide insight to characterize those who experience dysbiosis post-LEEP. Personalized medicine strategies are emerging as common therapies^65^ and may be a novel approach to take when treating patients who may not conform with the apparent restoration of a healthy FUT microenvironment post-LEEP.

Additionally, this study investigated whether these bacterial compositions could be associated with any symptomatic outcomes specifically related to the parameter of sexual functioning. Our data identified 1 participant (3) who had FSFI scores indicative of SD pre-LEEP; post-LEEP, this participant had improved FSFI scores above the threshold for SD. Decreases in proinflammatory and protective butyrate-producing bacteria (*Butyricicoccus* and *Intestinimonas*) were observed in this participant, possibly indicating a normalized healing response in the FUT. *Butyricicoccus* has been shown to restore cellular damage that results from cytokine-induced inflammation responses,^66^ and *Intestinimonas* has been shown to reduce proinflammatory signals.^56^ Both these protective bacteria have been investigated as novel probiotic treatments^66^^,^^67^ and could be considered in the future as possible interventions^66^ for this patient population with CD. Our findings also showed that 2 participants (20 and 23) had increased inflammatory bacteria post-LEEP. Interestingly, both had FSFI scores indicating SD before and after treatment. This may suggest that persistent or increased FUT dysbiosis could be associated with decreases in sexual functioning. It has been shown that some women with dysbiosis in the cervicovaginal microenvironment can develop symptoms such as vaginal discharge and inflammation and conditions such as bacterial vaginosis.^6^^,^^47^ As well, there is evidence that women with certain vaginal community state types (ie, CST IV) can have high production of inflammatory cytokines, which can cause epithelial damage and facilitate disease progression.^68^^,^^69^ While this detailed immunological and bacterial classification was not completed in the present study, it is possible that some women may have chronic inflammation that appears to persist post-LEEP. Mechanisms that link SD symptoms to chronic inflammation have been studied in the gut^70^ but not in the FUT and should be explored to design therapeutic treatment options. In men, however, inflammatory bacteria have been associated with prostatitis and SD.^71^ Targeted treatments have proved efficacious in reducing inflammation and subsequently improving SD symptoms,^71^ and similar considerations should be given to patients with CD who have these issues.

### Clinical implications

It is possible that CD itself, likely as a result of HPV infection, initiates an inflammatory response in the FUT, including the regions of the cervix, vagina, and urethra. The bacteria involved in either the disease pathogenesis or the innate immune response appear to be related to decreases in the sexual functioning domains in some patients. That is, the dysbiosis present in the FUT at the time of CD before treatment may contribute to impaired cellular functions that manifest as physical symptoms, in this case related to sexual functioning. Our results show that CD treatment by LEEP mostly restores microenvironment dysbiosis in the FUT. However, patients do not show a uniform healing response. It may be pertinent to reflect on the individual treatment of patients, as it seems that not all have the same microenvironment restoration post-LEEP. Some bacteria investigated in this study are currently being considered as therapeutic probiotics for inflammatory conditions. Future investigations should consider evaluating CD patients as candidates for these therapeutic treatments, for relief of persistent dysbiosis and any related SD symptoms.

### Strengths and limitations

This is the first study to investigate relationships among the cervical, vaginal, and urinary regions composing the FUT. We also utilized standard clinical examinations and validated questionnaires to allow for reproducibility. Importantly, this study took a novel approach to investigate associations of CD microbiota and sexual functioning, including at the level of individual participant microbiota profiles. This study also has several limitations. COVID-19 pandemic restrictions limited participant recruitment and increased participants lost to follow-up. The small sample size reduces the power of interpretation. As well, this study performed genus- rather than species-level bacterial analysis, which limits specificity of the findings. We were further unable to control for variables that could affect microbiota composition, though these data were collected and reported in participant demographics. Last, this study did not evaluate the psychological factors of a CD diagnosis, which could contribute to SD symptoms and self-report FSFI scores. Rather, our study evaluated the physiologic microbiota components and how they might contribute to self-reported SD.

## Conclusion

Our data provide evidence that there are differences in bacterial communities among the cervix, vagina, and urethra, and we hypothesize that these genera may play a role in the resolution or persistence of CD dysbiosis post-LEEP. Probiotic therapies could be a novel avenue of treatment for those who experience bothersome symptoms specifically related to sexual functioning that can be attributed to an inflammatory FUT microenvironment. This preliminary work contributes to emerging and ongoing evidence in the field and could lead to the development of studies designed to distinguish bacteria that correlate with SD symptoms, with the aim of restoring FUT homeostasis in patients with CD after treatment with LEEP.

## Funding

This study was funded by the ISSWSH Scholars in Women’s Sexual Health Research Grant.

## Conflicts of interest

None declared.

## Data availability

The data that support the findings of this study are available from the corresponding author, OG, upon reasonable request.
